# The feasibility, repeatability, validity and responsiveness of the EQ-5D-3L in Krio for patients with stroke in Sierra Leone

**DOI:** 10.1186/s12955-024-02246-x

**Published:** 2024-03-28

**Authors:** Daniel Youkee, Sahr Pessima, Catherine Sackley, Marina Soley-Bori, Gibrilla F. Deen, Iain J. Marshall

**Affiliations:** 1 King’s School of Life Course and Population Sciences, King’s College London, London, UK; 2 College of Medicine and Allied Health Sciences, University of Sierra Leone, Freetown, Sierra Leone; 3 School of Medicine and Rehabilitation, University of Nottingham, Nottingham, UK

**Keywords:** Stroke, EQ-5D, Quality of life, Sierra Leone, Africa, Psychometric

## Abstract

**Objectives:**

To assess the feasibility, repeatability, validity and responsiveness of the EQ-5D-3L in Krio for patients with stroke in Sierra Leone, the first psychometric assessment of the EQ-5D-3L to be conducted in patients with stroke in Sub Saharan Africa.

**Methods:**

A prospective stroke register at two tertiary government hospitals recruited all patients with the WHO definition of stroke and followed patients up at seven days, 90 days and one year post stroke. The newly translated EQ-5D-3L, Barthel Index (BI), modified Rankin Scale (mRS) and National Institute of Health Stroke Scale (NIHSS), a measure of stroke severity, were collected by trained researchers, face to face during admission and via phone at follow up. Feasibility was assessed by completion rate and proportion of floor/ceiling effects. Internal consistency was assessed by inter item correlations (IIC) and Cronbach’s alpha. Repeatability of the EQ-5D-3L was examined using test–retest, EQ-5D-3L utility scores at 90 days were compared to EQ-5D-3L utility scores at one year in the same individuals, whose Barthel Index had remained within the minimally clinical important difference. Known group validity was assessed by stroke severity. Convergent validity was assessed against the BI, using Spearman’s rho. Responsiveness was assessed in patients whose BI improved or deteriorated from seven to 90 days. Sensitivity analyses were conducted using the UK and Zimbabwe value sets, to evaluate the effect of value set, in a subgroup of patients with no formal education to evaluate the influence of patient educational attainment, and using the mRS instead of the BI to evaluate the influence of utilising an alternative functional scale.

**Results:**

The EQ-5D-3L was completed in 373/460 (81.1%), 360/367 (98.1%) and 299/308 (97.1%) eligible patients at seven days, 90 days and one year post stroke. Missing item data was low overall, but was highest in the anxiety/depression dimension 1.3% (5/373). Alpha was 0.81, 0.88 and 0.86 at seven days, 90 days and one year post stroke and IIC were within pre-specified ranges. Repeatability of the EQ-5D-3L was moderate to poor, weighted Kappa 0.23–0.49. EQ-5D-3L utility was significantly associated with stroke severity at all timepoints. Convergent validity with BI was strong overall and for shared subscales. EQ-5D-3L was moderately responsive to both improvement Cohen’s D 0.55 (95% CI:0.15—0.94) and deterioration 0.92 (95% CI:0.29—1.55). Completion rates were similar in patients with no formal education 148/185 (80.0%) vs those with any formal education 225/275 (81.8%), and known group validity for stroke severity in patients with no formal education was strong. Using the Zimbabwe value set instead of the UK value set, and using the mRS instead of the BI did not change the direction or significance of results.

**Conclusions:**

The EQ-5D-3L for stroke in Sierra Leone was feasible, and responsive including in patients with no formal education. However, repeatability was moderate to poor, which may be due to the study design, but should add a degree of caution in the analysis of repeated measures of EQ-5D-3L over time in this population. Known group validity and convergent validity with BI and mRS were strong. Further research should assess the EQ-5D in the general population, examine test–retest reliability over a shorter time period and assess the acceptability and validity of the anxiety/depression dimension against other validated mental health instruments. Development of an EQ-5D value set for West Africa should be a research priority.

**Supplementary Information:**

The online version contains supplementary material available at 10.1186/s12955-024-02246-x.

## Background

Stroke is the third leading cause of death and disability worldwide [1] and significantly impacts the health related quality of life (HRQoL) [2] of stroke survivors. Currently, little is known about HRQoL after stroke in African populations [3] and the EQ-5D has not been validated for patients with stroke in Sub Saharan Africa (SSA). A systematic review in 2020 of HRQoL after stroke in Africa found 28 studies conducted in 8 countries [3]. The review reported that only 4 (14.3%) of the 28 included studies used a translated, adapted instrument and only 3 (10.7%) used a psychometrically tested instrument [3]. To advance the measurement of HRQoL in stroke survivors in Sierra Leone, in this paper we assess the EQ-5D-3L translated into Krio.

The EQ-5D is the most widely used generic HRQoL instrument [4]. The EQ-5D-3L is a simple, generic, preference-based measure for health that can be used in clinical and economic evaluations [4], originally created by a multilingual group of researchers from five European countries [5]. The EQ-5D-3L describes HRQoL through five dimensions; mobility; self-care; usual activities; pain/discomfort and anxiety/depression, each containing 3 levels; no problem, moderate problem or severe problem, and a visual analogue scale from 0–100 with 100 indicating the best health imaginable and 0 the worst health imaginable. The EQ-5D has been used to measure HRQoL in patients with stroke for over 20 years [6]. The EQ-5D has been demonstrated to have reasonable validity and reliability, in geographically diverse stroke populations [7–10]. To date, the performance of the EQ-5D has been evaluated in Africa but only in non-stroke populations; for Xhosa in South Africa [11]; Yoruba in Nigeria [12]; Amharic in Ethiopia [13] and Chichewa in Malawi [14]. The Xhosa version was concluded to be reliable and valid, although concern was raised about the conceptual equivalence of the anxiety and depression dimension [11]. The Yoruba version of the EQ-5D-5L had acceptable reliability but demonstrated poor convergent validity with the 12-item Short-Form Health survey [12]. The Amharic version demonstrated good reliability and strong known group validity in adolescents with mental health disorders in Ethiopia [13]. The Chichewa version demonstrated good reliability and moderate convergent validity with the World Health Organization Quality of Life questionnaire [14]. In this publication we aim to validate the EQ-5D-3L for the first time in a stroke population in Africa.

Our research group translated the EQ-5D-3L into Krio for Sierra Leone, for use in the Stroke in Sierra Leone (SISLE) prospective longitudinal stroke register [15]. In this paper we use SISLE registry data to investigate the feasibility, repeatability, validity and responsiveness of the newly translated instrument.

## Methods

### Cohort

A prospective stroke register was established at the two principal adult tertiary government hospitals in Freetown, Sierra Leone at Connaught Teaching Hospital from 1st May 2019 until 30th September 2021 and at 34th Military Hospital from 1st February 2021 until 2nd September 2021. All consecutive patients 18 years and over meeting the WHO definition of stroke were included. The study methods and the health care setting have been previously described in depth [15]. Data were collected on admission, at seven days post stroke face to face, and via phone at 90 days post stroke, and one year post stroke. The EQ-5D-3L was introduced for follow up on the 24th February 2020 and at seven days post stroke on the 22nd June 2020. During admission, interviews were conducted at a private space close to the patient’s bedside, follow up interviews were conducted by telephone [16] by research assistants who were native Krio speakers and trained in Good Clinical Practice [17]. Sociodemographic details were recorded in an interview with the patient and caregiver. Educational attainment was classified as; no school; completion of primary school; completion of Basic Education Certificate Examination (BECE); completion of West African Senior school certificate examination (WASSCE); graduate degree; and Masters degree or higher. A binary variable for higher educational attainment was created, with the cut-off as completion of high school (WASSCE). Educational level is a useful proxy of socioeconomic status in our setting, due to the difficulties in measuring household income due to most individuals working in the informal economy. Patients were recorded as being resident in the Western Area, the catchment area of the two hospitals and the capital Freetown, or as resident outside of the Western Area, the remaining districts in the country. If the patient was unable to answer the EQ-5D-3L questions, a proxy response was recorded from the principal caregiver.

Responses were recorded on paper copies of the CRF, double data entry was conducted, and all data uploaded onto REDCap™.

### Instruments

The EQ-5D-3L Interviewer Administered v2.1 was translated into Krio for Sierra Leone using an expanded methodology based on the EuroQol Foundation guidelines [18] including focus groups, a process of iterative forward and backward translation, two independent forward translations and two independent back translations, followed by review and reconciliation. EQ-5D-3L was selected instead of the EQ-5D-5L due to difficulties of translating the five levels into Krio and difficulties in discriminating between the five levels with similar issues experienced by other researchers [19]. The translated tool can be accessed here [20]. EQ-5D health state responses can be converted into a single summary score, using a value set, which measures people’s preferences with respect to health. Value sets vary as different cultures may ascribe different values to individual health states. This poses a challenge to researchers in West Africa, as there is no EQ-5D value set for Sierra Leone or any country in West Africa [21], although recent value sets for the 5 level version (EQ-5D-5L) as opposed to the 3 level version (EQ-5D-3L) have been created for Ethiopia and Uganda. We therefore used the UK EQ-5D-3L value set in the main analysis because; it was generated using a large representative sample size [22]; there are linguistic links between English and Krio and the source questionnaire for our translation was in English. We present a sensitivity analysis conducted using the Zimbabwe EQ-5D-3L value set, the only SSA EQ-5D-3L value set, in the Supplementary Material to assess the impact of different value sets on the main results.

The stroke register also included the following measures. Functional status was measured by the ten item Barthel Index (BI) [23], scored from 0–100, translated by the study team into Krio, using a simple forward translation method. Disability was measured using the modified Rankin scale (mRS) from 0–6 [24]. Stroke severity was measured by National Institute of Health Stroke Scale (NIHSS) [25], by trained clinicians, as a continuous variable and was further categorised into mild stroke (NIHSS < 8), moderate stroke (NIHSS 8–15) and severe stroke (NIHSS > 15).

### Analysis

All data were transcribed onto written case report forms. Double data entry into RedCap was conducted. Statistical analyses were performed in STATA v17, StataCorp™ [26]. As the degree of missingness in the data was low (maximum of 1.3% across variables), we used complete case analysis. First the EQ-5D-3L, BI, mRS and NIHSS response distributions were tested for normality using scatterplots, comparison of mean, median and Shapiro test. The feasibility, repeatability, validity and responsiveness of the EQ-5D-3L in Krio was evaluated using various methods see Table 1.

**Table 1:** Properties of the Krio EQ-5D-3L assessed, statistics used, pre-specified values and data timepoint used

Property assessed	Statistic used	Pre-specified values	Timepoint used
**Construct validity**			
Feasibility	Completion rate % missing item data	< 5% [27]	Seven days, 90 days, one year
Floor effects Ceiling effects	% of patients with the lowest possible score or highest possible score	significant if ≥ 15%, moderate if 10% to < 15%, minor if 5% to < 10%, and negligible if < 5%	Seven days
Internal consistency	Inter item correlation	0.15–0.75: suitable [28]	Seven days, 90 days and one year
	Cronbach’s alpha	0.6–0.7: acceptable 0.8–0.9: good [29]	Seven days, 90 days and one year
**Repeatability**			
Repeatability Test–retest	Weighted Kappa	very poor = < 0.20: Poor = 0.20 -0.39; moderate = 0.40–0.59; Good: 0.60—0.79, excellent = : 0.80 – 1.0	90 days compared to one year
**Known Group Validity**			
Known group validity for stroke severity	Kruskal Wallis	*p* < *0.05*	Seven days, 90 days, one year
**Convergent Validity**			
Convergent validity with BI	Spearman’s correlation coefficient	moderate > 0.4, > 0.5 strong	Seven days, 90 days, one year
	Spearman’s rho	0.20 and 0.35 were considered weak, between 0.35 and 0.50 moderate and > 0.50 strong [30]	90 days
**Responsiveness**			
Responsiveness MCID BI > 9.25 points	Cohen’s (D) Effect sizes	< 0.20 trivial, small 0.20–0.50, moderate 0.50–0.80 or large > 0.80	Seven days and 90 days

*BI* Barthel Index, *MCID* Minimal clinically important difference

Repeatability of the EQ-5D-3L was examined using test–retest. EQ-5D-3L utility scores at 90 days were compared to EQ-5D-3L utility scores at one year in the same individuals, whose Barthel Index had remained within the minimally important clinical difference (± 9.25 points) for patients with stroke [31]. Due to the long duration from test to retest we expected some change in EQ-5D-3L scores. Content validity for the EQ-5D-3L was explored during the translation process. Known groups validity is determined by the degree to which an instrument demonstrates different scores, for pre-specified hypotheses, for groups known to vary on the variables being measured. Our study hypothesises that patients with more severe stroke will have lower EQ-5D-3L utility scores than those with less severe stroke. We analyse EQ-5D-3L utility scores in patients with mild, moderate and severe stroke, measured by NIHSS on admission [32, 33]. Kruskal Wallis test was used to compare EQ-5D-3L utility scores by stroke severity (mild, moderate and severe).

We hypothesised that there would be strong correlation between BI score and EQ-5D-3L utility score at seven days, 90 days and one year [34]. Scatterplots were created and Spearman’s correlation coefficient was calculated to assess correlation at 90 days. We hypothesised that there would be*,* moderate or strong correlation between EQ-5D-3L dimensions and BI items of the same dimension, and lower levels of correlation between EQ-5D-3L dimensions which are not measured by the BI [35]. We utilised the conceptual model developed by Kaambwa et al [35], to map EQ-5D-3L dimensions to Barthel Index items. The conceptual model mapped two EQ-5D-3L dimensions to eight BI items: EQ-5D-3L dimension ‘Mobility’ mapped to BI items “Mobility’, ‘Stairs’ and ‘Transfer’; EQ-5D-3L dimension of ‘Self-Care’ mapped to BI items ‘Bathing’, ‘Grooming’, ‘Feeding’, ‘Dressing’ and ‘Toilet use’. We assessed correlation between EQ-5D-3L dimensions and BI items, using Spearman’s rho.

Responsiveness was examined by assessing EQ-5D-3L utility scores at seven days compared to EQ-5D-3L utility scores at 90 days in individuals whose Barthel Index had increased or decreased by the minimally clinical important difference (> 9.25 points) (MCID) [31] and whose EQ-5D-3L scores were not at ceiling at seven days.

Three sensitivity analyses were conducted to evaluate their impact on psychometric performance of the EQ-5D-3L:

1. To evaluate the influence of utilising an alternative EQ-5D-3L value set, we conducted a sensitivity analysis using the Zimbabwe value set.
2. To evaluate the influence of utilising an alternative functional scale, we conducted a sensitivity analysis using mRS in place of BI.
3. To evaluate the influence of patient educational attainment, we conducted a sensitivity analysis including only patients with no formal education.

The study is reported using the COSMIN reporting checklist [36].

## Results

The sociodemographic, comorbidities, NIHSS, BI and stroke type of patients in the cohort at 90 days and one year is shown in Table 2.

**Table 2:** Descriptive univariable statistics of cohort at 90 days and one year, count (%) unless specified

	90 days *N* = 360	One year *N* = 300
Mean Age (SD)	57.2 (SD: 14.1)	55.7 (SD: 13.1)
Male sex	182 (50.6%)	145 (48.3%)
Higher educational attainment	146 (41.0%)	122 (41.5%)
Employed	148 (43.1%)	135 (46.7%)
Prior stroke	36 (9.8%)	29 (9.7%)
Resident Western Area	289 (79.2%)	251 (83.7%)
Hypertension	312 (85.3%)	259 (86.3%)
Diabetes	67 (18.3%)	60 (20.0%)
Mean NIHSS (SD)	11.8 (SD: 7.2)	11.7 (SD: 7.0)
Mean Post-stroke BI (SD)	41.9 (SD 27.4)	40.5 (SD 28.1)
Stroke type		
Ischaemic	279 (76.2%)	224 (74.7%)
Intracerebral haemorrhage	75 (20.5%)	65 (21.7%)
Subarachnoid haemorrhage	5 (1.4%)	6 (2.0%)
Undetermined	7 (1.9%)	5 (1.7%)

*SD* Standard Deviation, *NIHSS* National Institute of Health Stroke Scale, *BI* Barthel Index

The EQ-5D-3L was completed on 373/460 (81.1%) eligible patients at seven days post stroke, on 360/367 (98.1%) eligible patients at 90 days post stroke and 299/308 (97.1%) patients at one year post stroke, see Table 1*.* At seven days, patients who did not complete the EQ-5D-3L had more severe strokes and higher rates of aspiration pneumonia compared to those who completed the EQ-5D-3L, at 90 days and one year there was no significant differences between the completion and non-completion groups. Completion rates at seven days were similar between patients with no formal education and 148/185 (80.0%) and with any formal education 225/275 (81.8%)*.* Proxy reporting was highest in the acute phase of stroke, 103/373 (27.6%) at seven days then 49/360 (13.6%) at 90 days and 47/308 (15.3%) at one year. Case fatality at seven days, 90 days and one year was 37%, 44% and 49% respectively, and is reported in detail elsewhere [37]. Follow up rates were 88.3% at 90 days and 81.5% at one year for participants alive at last point of contact [15]. Patients lost to follow up were more likely to be male, resident outside of the Western Area and had a lower stroke severity measured by NIHSS [15].

Missing item data was low at all timepoints and was highest 0.5% (11/2268) at seven days post stroke. The EQ-5D-3L dimension with the highest missing data 1.3% (5/373) was the anxiety and depression dimension at seven days post stroke, Table 3. Floor and ceiling effects were minor. At baseline, seven days post stroke 28/37 (7.5%) 3 had the highest possible score 1, 25/373 (6.7%) patients had the lowest possible score -0.594. The number of patients with the lowest possible EQ-5D-3L utility value was 25 (6.7%) at seven days, 6 (1.7%) at 90 days and 2 (0.9%) at one year. The number of patients with the highest possible EQ-5D-3L utility value was 28 (7.5%), 133 (36.9%) at 90 days and 80 (36.7%) at one year.

**Table 3:** EQ-5D-3L health states, response rate, missing item data and visual analogue scales at seven days, 90 days, one year post stroke

	**Count N**	**Missing N (%)**	**No difficulty N (%)**	**Moderate difficulty N (%)**	**Severe difficulty N (%)**
***Seven days post stroke n* = *378***					
Mobility	378	0	86 (22.8)	138 (36.5)	158 (40.7)
Self-care	377	1	73 (19.4)	155 (41.4)	149 (35.2)
Activities	377	1	44 (11.7)	127 (33.7)	206 (54.6)
Pain	375	3	127 (33.9)	185 (49.3)	63 (16.8)
Anxiety	373	5	174 (46.7)	151 (40.5)	48 (12.9)
VAS	377	1	Mean 46.1 (SD:21.2)		
***90 days post stroke n* = *362 out of 367 contacted (98.6%)***					
Mobility	362	0	218 (60.2)	117 (32.3)	27 (7.5)
Self-care	362	0	214 (59.1)	115 (31.8)	33 (9.1)
Activities	362	0	167 (46.1)	107 (29.6)	88 (24.3)
Pain	362	0	193 (53.3)	143 (39.5)	26 (7.2)
Anxiety	362	0	252 (69.6)	87 (24.0)	23 (6.4)
VAS	360	1	Mean 66.0 (SD:21.7)		
***One year post stroke n* = *300 out of 308 contacted (97.4%)***					
Mobility	300	0	166 (55.3)	115 (38.3)	19 (6.3)
Self-care	300	0	169 (56.3)	109 (36.3)	22 (7.3)
Activities	300	0	139 (46.3)	87 (29.0)	74 (24.7)
Pain	300	0	186 (62.0)	94 (31.3)	20 (6.7)
Anxiety	299	1	227 (75.9)	53 (17.7)	19 (6.4)
VAS	299	1	Mean 71.0 (SD:22.0)		

*VAS* Visual Analogue Scale

Median BI at seven days was 30 (IQR: 0–30), at ninety days 90 (IQR: 55–100) and at one year 95 (IQR: 62.5–100).

### Internal consistency

Cronbach’s alpha was 0.81, 0.88 and 0.86 at seven days, 90 days and one year post stroke respectively, and average interitem covariance 0.265. Inter-item correlations ranged from 0.46–0.74 and all were within our pre-specified range of 0.15–0.75, see Table 4*.*

**Table 4:** Inter-item correlation matrix for EQ-5D-3L at 90 days

	Mobility	Self-care	Activities	Pain	Anxiety
Mobility	1.000				
Self-care	.742	1.000			
Activities	.585	.635	1.000		
Pain	.614	.614	.547	1.000	
Anxiety	.636	.578	.461	.606	1.000

### Repeatability

Repeatability was assessed on 124 patients who had a BI at one year that was within ± 9.25 points of the BI score at 90 days and were classified as “stable” and included in the analysis.. Weighted Kappa was poor for mobility (0.36) usual activities (0.23), pain (0.23) and anxiety/depression (0.23) and moderate for self-care (0.49), as per the prespecified cut-offs.

#### Known group validity

EQ-5D-3L utility was significantly associated with stroke severity, Kruskal–Wallis test at seven days post stroke chi2 = 72.1 (*p* =  < 0.001) at 90 days Chi2 = 17.2 (*p* =  < 0.001), and at one year Chi2 = 20.6 (*p* =  < 0.001) see Table 5. Known groups validity was similar using the Zimbabwe value set and in patients with no formal education, see Supplementary Material.

**Table 5:** Known group validity of EQ-5D-3L values by stroke severity measured by NIHSS, significance test: Kruskal–Wallis

Stroke severity	EQ-5D-3L seven days median (IQR)	EQ-5D-3L 90 days median (IQR)	EQ-5D-3L One year median (IQR)
Mild stroke	0.56 (0.26–0.78)	0.87 (0.59–1.0)	1.0 (0.59–1.0)
Moderate stroke	0.17 (-.06–0.71)	0.69 (0.33–1.0)	0.66 (0.38–1.0)
Severe stroke	-0.06 (-0.33–0.26)	0.59 (0.19–1.0)	0.71 (0.31–1.0)
	chi2 = 72.1 *p* = < 0.001	Chi2 = 17.2 *p* = < 0.001	Chi2 = 20.6 *p* = < 0.001

Scatter plots of Barthel Index and EQ-5D-3L utility value demonstrated higher correlation at 90 days (*R*^2^ = 59.8%) and one year (*R*^2^ = 59.4%) compared to seven days post stroke (*R*^2^ = 26.6%). Spearman’s rho between BI and the EQ-5D-3L was moderate at seven days 0.49 and strong at 90 days 0.72 and one year 0.79*.* Sensitivity analysis using the Zimbabwe value set showed overall higher correlation between BI and EQ-5D-3L compared to the UK value set, with a similar pattern of correlations being lower at seven days, higher at 90 days and highest at one year post stroke.

#### Convergent validity of EQ-5D dimensions and BI items

Convergent validity of the EQ-5D-3L dimensions compared to the BI items is shown in Table 6.

**Table 6:** Spearman’s rho between EQ-5D-3L dimensions and BI items at 90 days post stroke

	EQ-5D dimension				
Barthel Index dimension	Mobility	Self-care	Usual activities	Pain	Anxiety/depression
Feeding	-0.52	**-0.58**^**a**^	-0.43	-0.48	-0.56
Bathing	-0.61	**-0.69**^**a**^	-0.53	-0.5	-0.53
Grooming	-0.54	**-0.63**^**a**^	-0.49	-0.46	-0.53
Dressing	-0.72	**-0.78**^**a**^	-0.6	-0.56	-0.53
Bowels	-0.5	**-0.54**^**a**^	-0.42	-0.38	-0.58
Bladder	-0.49	-0.47	-0.39	-0.41	**-0.57**^**a**^
Toilet	**-0.67**^**a**^	-0.64	-0.48	-0.56	-0.57
Transfers	**-0.75**^**a**^	-0.73	-0.57	-0.55	-0.57
Mobility	**-0.75**^**a**^	-0.72	-0.57	-0.57	-0.57
Stairs	**-0.69**^**a**^	-0.65	-0.43	-0.5	-0.52

^a^Denotes the highest correlation value in the column to identify the highest correlations between BI items and EQ-5D-3L dimensions

The highest correlations for transfers, mobility and stairs were for the mobility dimension of the EQ-5D-3L. The highest correlations for bathing, grooming, dressing were for the self-care dimension of the EQ-5D-3L. Toilet use had a higher correlation -0.6705 to mobility than to self-care -0.6418. The highest correlation for “Bladder problems and associated urinary incontinence” item was to the anxiety/depression dimension.

#### Responsiveness

Responsiveness was measured in 195 patients who had a MCID in the BI from seven days to 90 days post stroke, see Table 7*.*

**Table 7:** Responsiveness of EQ-5D-3L shown by Median EQ-5D-3L at seven days and 90 days, disaggregated by improved patients BI = Improved by > 9.25, stable patients and patients whose functional level decreased by > 9.25

Barthel Index	Number	Median (IQR) EQ-5D-3L seven days	Median (IQR) EQ-5D-3L 90 days	Cohen’s D	95% CI
Patients with improved function (Increased by > 9.25)	178	0.31 (-0.04–0.59)	0.78 (0.52–1.0)	.55	0.15—0.94
Stable	29	0.47 (0.14–0.85)	0.57 (.04–1.0)		
Patients with lower function (decreased by > 9.25)	17	0.59 (0.19–0.71)	0.19 (-0.16–0.52)	0.92	0.29—1.55

For patients whose functional level increased, compared to patients whose functional level remained stable, EQ-5D-3L utility score was statistically higher *p* = *0.008*, for patients whose functional level decreased EQ-5D-3L utility score was significantly lower, *p* = *0.004*.

## Discussion

This study presents psychometric validation of the EQ-5D-3L in Krio for patients with stroke in Sierra Leone, the first validation of EQ-5D for patients with stroke in Africa and adds to the small number of EQ-5D validation studies conducted in any populations in Africa [11–14].

The feasibility and acceptability of the EQ-5D-3L was good, response rates were high and missing item data were low compared to objective standards [27, 38]. Completion rates were similar in patients with any formal education compared to patients with no formal education. We believe this is because we used an interviewer administered EQ-5D-3L, as opposed to the self-complete [39]. The lowest response rate of 82.2% was at seven days post stroke and may reflect that interviewers were trained not to initiate or continue interviews if there were signs of patient emotional distress, which may have been higher in the immediate post stroke period relative to 90 days or one year. The highest number of missing items was in the anxiety and depression dimension, this dimension also had the highest proportion of ceiling effects at all three timepoints. This may indicate acceptability issues with questions of mental wellbeing, or reflect findings from our translation process, which found the EQ-5D-3L anxiety and depression dimension the hardest to translate into Krio, with similar findings reported in the Xhosa version of the EQ-5D [11].

Repeatability of the EQ-5D-3L was moderate to poor, this may be due to the study design with a long period between test and re-test, but should add a degree of caution in the analysis of repeated measures of EQ-5D-3L over time in this population. Internal consistency was good, with all inter item correlations within our pre-specified range and Cronbach’s alpha from 0.81–0.86. Given that the EQ-5D-3L is only a five-item instrument, with no subscales, it is also unlikely that the high alpha value is driven by redundancy in the instrument. Additionally, we tested whether the high Cronbach’s alpha is being driven by variance in our sample, alpha remained between 0.839–0.888 when disaggregated into mild stroke, moderate stroke and severe stroke. Cronbach’s alpha was similar for patients with no formal education, we conclude that this is evidence of strong internal consistency of the Interviewer Administered EQ-5D-3L in our population, inclusive of patients with no formal education.

We report strong known groups validity of the EQ-5D-3L, similar to other studies describing known group validity with stroke severity [40, 41]. Similar to others we demonstrate strong convergent validity between BI and EQ-5D-3L for patients with stroke [9]. Convergent validity was lower at seven days post compared to 90 days and one year post stroke. Convergent validity was highest across shared subscales of the two instruments and lowest for non-shared subscales, pain/discomfort and anxiety depression, similar to results presented by others [35, 42]. Further research should examine convergent validity of EQ-5D-3L pain/anxiety and depression/anxiety dimensions against culturally adapted and tested pain and mental health instruments in Sierra Leonean populations.

We found EQ-5D-3L to be moderately responsive to both improvement and deterioration. Our effect sizes were similar to a study in Canadian patients with stroke [43] and higher than a study in German patients with stroke [44]. Our sensitivity analyses, using the mRS as an external anchor for responsiveness, demonstrated moderate to small effect sizes. Predictably, effect size varied from small, moderate to large, depending on the degree of improvement and instrument used to measure improvement. Finally, we relied on a MCID from a study of stroke survivors in Taiwan [7], whereas the MCID for stroke survivors in Sierra Leone may differ.

Overall, results were consistent between the analyses conducted using the UK and Zimbabwe value sets, with slightly stronger correlations between EQ-5D-3L and BI using the Zimbabwe value set. Feasibility, reliability, validity and responsiveness results using the mRS in place of BI produced similarly results. Importantly, in a country with adult literacy rates of 47.7% [45], subgroup analysis of those with no formal education produced similar results.

### Limitations

The duration between follow ups, necessitated by the design of the stroke register to assess repeatability, was too long, and we therefore had to use the BI as an anchor. Further work should assess repeatability across a shorter time period. In our study design the BI and the EQ-5D-3L were administered by the same researcher during the same interview, ideally the EQ-5D-3L should have been administered by a researcher blind to the BI results, however our study design did not permit this. Furthermore, the BI was translated using a simple forward translation process into Krio, rather than a thorough translation and adaption as per the EQ-5D-3L. Although recent EQ-5D-5L value sets have been created in Ethiopia [46] and Uganda, our work is limited by the lack of a representative EQ-5D value set for countries in West Africa, which would be a timely and important piece of research. Finally, our cohort is drawn from a hospital-based register of patients who suffered severe strokes, rather than a population-based register. The stroke severity and case fatality rate in our cohort is at the high end of estimates in SSA [47] and should be accounted for when generalizing from these results.

### Strengths

We report the first validation of the EQ-5D in a stroke population in Africa. Our study has a large sample size and was conducted in a well-designed prospective stroke register, using standardized instruments and trained researchers [15]. Sensitivity analyses confirmed our findings including testing in patients with no formal education.

## Conclusion

In our study we report that the interviewer administered EQ-5D-3L in Krio for patients with stroke in Sierra Leone was feasible, reliable, and responsive including in patients with no formal education. The EQ-5D-3L demonstrated strong known groups validity by stroke severity and strong convergent validity with the BI, which was greater at 90 days and one year compared to seven days. Further research should examine repeatability of the instrument and assess the acceptability and validity of the anxiety/depression dimensions through comparison to culturally adapted mental health instruments. Our research is limited by the lack of a representative EQ-5D value set for West Africa and this should be an area for future research.

### Supplementary Information

**Supplementary Material 1.**

## Data Availability

Data from the SISLE register is available to other stroke researchers, and the SISLE researchers are interested in collaborating in more detailed and comparative research. Requests for data access for academic use should be made to the King’s College London (KCL) stroke research group where data will be made available subject to academic review and acceptance of a data-sharing agreement. Requests should be made by email to registry.comahs@usl.edu.sl and stroke-register@kcl.ac.uk. Request should include a 400 word scientific abstract, with the following titles: Introduction, Scientific rationale, Methods, Results and Potential impact of the research. Requests should be accompanied by cv of the principal researchers. Requests will be reviewed and decisions communicated by the KCL stroke research group, within 6 weeks of submission date.

## Abbreviations

BI: Barthel Index
HRQoL: Health related quality of life
IIC: Inter item correlations
MCID: Minimal clinically important difference
MRS: Modified Rankin Scale
NIHSS: National Institute of Health Stroke Scale
SISLE: Stroke in Sierra Leone
SSA: Sub Saharan Africa
