# Supplementary material for: The feasibility, repeatability, validity and responsiveness of the EQ-5D-3L in Krio for patients with stroke in Sierra Leone
Source: Health Qual Life Outcomes. 2024 Mar 28;22:29. doi: 10.1186/s12955-024-02246-x (PMC10976786; doi:10.1186/s12955-024-02246-x)
Supplement: Supplementary file 1 — Supplementary Material 1. [file 12955_2024_2246_MOESM1_ESM.docx]

**Supplementary Material**

**Contents**

1. ***Supplementary table one:*** *Cronbach’s alpha for EQ-5D-3L disaggregated by stroke severity measured by NIHSS, to demonstrate whether high alpha is driven by variance*
2. ***Sensitivity Analysis using Zimbabwe value set instead of UK value set***
3. ***Sensitivity analysis with Modified Rankin Scale in place of Barthel Index***
4. ***Sensitivity analysis 3: Subgroup analysis of patients with no formal education***

| Stroke severity | Cronbach’s alpha | Average interitem covariance |
| --- | --- | --- |
| Mild stroke | 0.839 | 0.169 |
| Moderate stroke | 0.881 | 0.287 |
| Severe stroke | 0.888 | 0.309 |

*Supplementary Table one: Cronbach’s alpha for EQ-5D-3L disaggregated by stroke severity measured by NIHSS, to demonstrate whether high alpha is driven by variance*

**Sensitivity Analysis using Zimbabwe EQ-5D-3L value set instead of UK value set**

**Known groups validity**

| Stroke severity | EQ-5D-3L seven days median (IQR) | EQ-5D-3L 90 days median (IQR) | EQ-5D-3L One year median (IQR) |
| --- | --- | --- | --- |
| Mild stroke | 0.65 (0.51-0.80) | 0.86 (0.70-1.0) | 1.0 (0.71-1.0) |
| Moderate stroke | 0.48 (0.32-0.64) | 0.77 (0.60-1.0) | 0.71 (0.60-1.0) |
| Severe stroke | 0.28 (0.09-0.55) | 0.67 (0.50-1.0) | 0.71 (0.57-1.0) |
|  | chi2=73.1 p=<0.001 | chi2=21.1 p=<0.001 | Chi2=23.8  p=<0.001 |

*Supplementary Table two: EQ-5D-3L values by stroke severity measured by NIHSS, using Zimbabwe value set.*

**Correlation with Barthel Index**

|  | **BI seven days** | **BI 90 days** | **BI one year** |
| --- | --- | --- | --- |
| **EQ-5D-3L seven days** | 0.52 |  |  |
| **EQ-5D-3L 90 days** |  | 0.72 |  |
| **EQ-5D-3L One year** |  |  | 0.85 |

*Supplementary Table three: Spearman’s Rho between EQ-5D-3L utility value and Barthel Index (BI) at seven days, 90 days and one year.*

R^2^  was 29.2%, 62.3% and 67.1% at seven days, 90 days and one year respectively.

**Sensitivity analysis with Modified Rankin Scale in place of Barthel Index**

| Modified Rankin Scale | Number | Median (IQR) EQ-5D-3L seven days | Median (IQR) EQ-5D-3L 90 days | Cohen’s D | 95% CI |
| --- | --- | --- | --- | --- | --- |
| Improved (>1 point decrease) | 206 | 0.49 (.08-0.66) | 1.0 (0.69-1.0) | 0.65 | -0.35-0.95 |
| Stable +/- 1 point | 136 | 0.26 (.002-0.66) | 0.52 (0.002-0.80) |  |  |
| Worsened (>1 point increase) | 7 | 0.27 (.20--.62) | 0.52 (-0.29-0.62) | 0.78 | -.78-1.03 |

*Supplementary Table four: Responsiveness of EQ-5D-3L utility values to changes in modified Rankin score from seven days to 90 days. EQ-5D-3L values for Improved mRS > 1 point decrease, Stable* +/- 1 point*, Worsened (*(>1 point increase*) from seven days to 90 days post stroke. *Cohen’s D thresholds were pre-specified as, <0.2 trivial, Small .20-.50, moderate 0.50-0.80 or large >0.80.*

For patients who had improved compared to stable patients EQ-5D-3L utility score was statistically higher *p=0.0002*, Mann Whitney U test. For patients who had worsened EQ-5D-3L utility score was not significantly lower, *p=0.74* Mann Whitney U test, note low sample size.

**Sensitivity analysis 3: Subgroup analysis of patients with no formal education**

Patients with EQ-5D-3L response and no formal education were 148/373 (39.7%) at seven days, 137/360 (38.1%) at 90 days and 105/299 (35.1%) at one year.

Completion rates at seven days were similar between patients with no formal education and 148/185 (80.0%) and with any formal education 225/275 (81.8%).

|  | Mobility | Self-care | Activities | Pain | Anxiety |
| --- | --- | --- | --- | --- | --- |
| Mobility | 1.000 |  |  |  |  |
| Self-care | 0.769 | 1.000 |  |  |  |
| Activities | 0.581 | 0.656 | 1.000 |  |  |
| Pain | 0.531 | 0.632 | 0.543 | 1.000 |  |
| Anxiety | 0.659 | 0.601 | 0.454 | 0.614 | 1.000 |

*Supplementary table five: Inter-Item Correlation Matrix for EQ-5D-3L at 90 days*

At 90 days post stroke, Cronbach’s alpha was 0.88 and average interitem covariance 0.30.

Inter-item correlations ranged from 0.454-0.769 and the covariance between Mobility and self-care was outside our pre-specified range of 0.15-0.75.

34 patients had a BI at one year that was within +/-9.25 points of the BI score at 90 days and were classified as “stable” and included in the analysis. Weighted Kappa was moderate for mobility (0.39), good for self-care (0.62), and poor for usual activities (0.10), pain (0.17) and anxiety/depression (0.19) as per the prespecified cut-offs.

**Known Group validity**

| Stroke severity | EQ-5D-3L seven days median (IQR) | EQ-5D-3L 90 days median (IQR) | EQ-5D-3L One year median (IQR) |
| --- | --- | --- | --- |
| Mild stroke | 0.52 (0.12-0.65) | 0.76 (0.56-1.0) | 1.0 (0.36-1.0) |
| Moderate stroke | 0.23 (0.01-0.54) | 0.56 (0.08-1.0) | 0.57 (0.32-0.80) |
| Severe stroke | -0.10 (-0.43-0.15) | 0.59 (0.01-1.0) | 0.52 (0.20-1.0) |
|  | chi2=21.3 p=<0.001 | Chi2=5.7  p=0.05 | Chi2=7.5  p=0.02 |

*Supplementary table six: Known group validity of EQ-5D-3L values by stroke severity measured by NIHSS, significance test: Kruskal-Wallis, for patients with no formal education.*

R^2^  was 26.2%, 64.1% and 67.1% at seven days, 90 days and one year respectively.

**Responsiveness**

| Barthel Index | Number | Median (IQR) EQ-5D-3L seven days | Median (IQR) EQ-5D-3L 90 days | Cohen’s D | 95% CI |
| --- | --- | --- | --- | --- | --- |
| Patients with improved function (Increased by >9.25) | 71 | 0.20 (-0.01-0.56) | 0.66 (0.43-1.0) | .82 | 0.03 – 1.6 |
| Stable | 7 | 0.52 (-.001-0.69) | 0.43 (-0.17-0.76) |  |  |
| Patients with lower function (decreased by >9.25) | 9 | 0.26 (0.08-0.80) | 0.08 (-0.16-0.59) | 0.25 | 1.2 – 0.75 |

*Supplementary Table seven: Responsiveness of EQ-5D-3L shown by Median EQ-5D-3L at seven days and 90 days, disaggregated by improved patients BI= Improved by >9.25, stable patients and patients whose functional level decreased by >9.25*
